# Supplementary material for: Competitive advantages of organizational project management maturity: A quantitative descriptive study in Australia
Source: PLoS One. 2023 Jun 27;18(6):e0287225. doi: 10.1371/journal.pone.0287225 (PMC10298749; doi:10.1371/journal.pone.0287225)

**Supplementary material**

**Appendix A**

**A list of various project management maturity models**

Capability Maturity Model for Software (SW-CMM)

Organizational Project Management Maturity Model (OPM3)

Change Proficiency Maturity Model

People Capability Maturity Model (PCMM)

Earned Value Management Maturity Model (EVM3)

Programme Management Maturity Model

eGovernment Maturity Model

Self-Assessment Maturity Model (SAMM)

ESI International’s Project Framework (ESI)

Software Reliability Engineering Maturity Model

IPMA Delta project management maturity model

**Appendix B**

**A List of General Maturity Models and Methods**

**
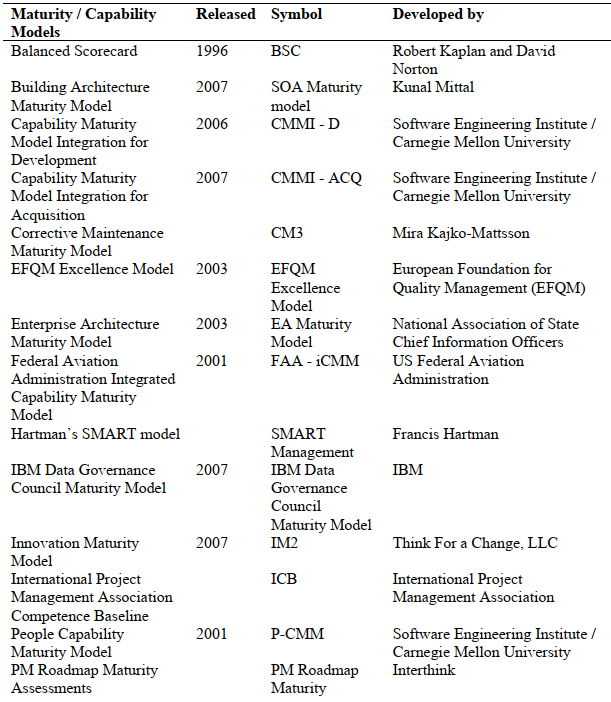
**

**Appendix C**

**Informed consent**

Dear Participant,

We are doing a research study entitled “Organizational project management maturity and its effects on competitive advantages”. The purpose of the research study is to determine the effects of organizational project management maturity level on competitive advantages in the Sydney based Australian project management consultancy industry.

Your participation will take approximately 20 minutes of your time and will involve answering a series of survey questions to determine your perception towards a series of scenarios. You can decide to be a part of this study or not. Once you start, you can withdraw from the study at any time without any penalty or loss of benefits. The results of the research study may be published but your identity will remain confidential and your name will not be made known to any outside party. The first 130 valid responses collected will be eligible to receive a Westfield gift card to the value of $30 (AUD).

In this research, there are no foreseeable risks to you.

Although there may be no direct benefit to you except for the gift card, a possible benefit from your participation in this study may provide a better understanding of the connection between organizational project management maturity and the associated competitive advantages. It is also possible to gain insight into the collective experiences of the frontline project managers on organizational project management maturity as well as the ability to make more informed strategic decisions by the senior organizational managers based on the organizational level of project management maturity on organizational strategies.

If you have any questions about the research study, please call me at 0415 XXX XXX or via email at XXXXX.XXXXX@optusnet.com.au For questions regarding the ethics approval process, please contact Bellberry HREC via email at XXXX@bellberry.com.au and quote protocol number 2016-06-508. For questions about your rights as a study participant, or any concerns or complaints, please contact the XXXXXX of XXXXX Institutional Review Board via email at XXX@phoenix.edu.

As a participant in this study, you should understand the following:

1. You may decide not to be part of this study or you may want to withdraw from the

study at any time including after the data is collected. If you want to withdraw, you

can do so without any penalty. In order to withdraw, a written request should be

directed to the email address of the researcher.

2. Your identity will be kept strictly confidential.

3. XXXXX XXXXX, the researcher, has fully explained the nature of the research study

and has answered all of your questions and concerns.

4. Data collected from the surveys will be stored in a digital format in a 128bit secure

folder. The data will be stored for a period of three years, and then destroyed.

5. The results of this study may be published.

By clicking on the radio button below that indicates “I AGREE”, you are indicating that you are over the age of 18 years old and give your permission to voluntarily serve as a participant in the study.

Please click one of the following and you will be taken to the survey questions:

( ) I accept the above terms. ( ) I do not accept the above terms

**Appendix D**

**Section 1: Project Management Maturity Model (P3M3®) self-assessment**

Please select the response that best describes the organization that you currently work for (or

for your most recent place of employment)

**Question 1**. Our organization can be best characterized as having:

(A) Processes that are not usually documented; there aren’t any/only a few process

descriptions. Actual practice is determined by events or individual preferences, and

performance is variable. Successful initiatives are often based on key individuals’ competencies rather than organization-wide knowledge and capability and the organization is unable to repeat past success consistently. Such “success” is often achieved with a budget and/or schedule overruns. Processes are undeveloped or incomplete. There is little, if any, guidance or supporting documentation, and even terminology may not be standardized across the organization – e.g. business cases, risks, issues, etc. may not be interpreted in the same way by all managers and team members.

(B) The organization can demonstrate that basic management practices have been

established – e.g. tracking expenditure and scheduling resources – and that processes

are developing. Some key individuals have had suitable training and who

can demonstrate a successful track record and through them, the organization is

capable of repeating earlier successes in the future.

Initiatives are performed and managed according to their documented plans; project

status and delivery is visible to management at defined points, such as on reaching

major milestones. The organization may still have inadequate measures of success; unclear

responsibilities for achievement; ambiguity and inconsistency in business objectives;

lack of fully integrated risk management; limited experience in change management;

and inadequacies in communications strategy.

(C) Management and technical processes are documented, standardized, and integrated to

some extent with other business processes. There is likely to be process ownership

and an established process group with responsibility for maintaining consistency and

delivering process improvements across the organization. Senior management is engaged consistently and provides active and informed support. There is likely to be an established training program to develop the skills and knowledge of individuals so they can more readily perform their designated roles. A key aspect of quality management will be the widespread use of peer reviews of identified products, to better understand how processes can be improved and thereby eliminate possible weaknesses.

A key distinction between this and the previous level description is the scope of

standards, process descriptions, and procedures. Processes will be managed more

proactively and the standard processes can be tailored to suit specific circumstances,

per explicit guidelines.

(D) The organization demonstrates mature behavior through **A List of General Maturity Models and Methods**defined processes that are

quantitatively managed – i.e. controlled using metrics and quantitative techniques.

There is good evidence of quantitative objectives for quality and process performance, and these are being used as criteria in managing processes. The measurement of data collected is contributing towards the organization’s overall performance measurement framework and facilitates portfolio analysis and ascertaining the current capacity and capability constraints. There is likely to be an established training programme to develop the skills and knowledge of individuals so they can more readily perform their designated roles. A key aspect of quality management will be the widespread use of peer reviews of identified products, to better understand how processes can be improved and thereby eliminate possible weaknesses. A key distinction between this and the previous level description is the scope of standards, process descriptions, and procedures. Processes will be managed more proactively and the standard processes can be tailored to suit specific circumstances, following explicit guidelines. Top management is proactively seeking out innovative ways to achieve goals. Using metrics, management can effectively control processes and identify ways to adjust and adapt them to particular initiatives without loss of quality.

(E) The organization is focused on the optimization of its quantitatively managed processes to take into account changing business needs and external factors. It can anticipate future capacity demands and capability requirements to meet delivery challenges – e.g. through portfolio analysis. Top managers are seen as exemplars, reinforcing the need and potential for capability and performance improvement. The knowledge gained by the organization from its process and product metrics will enable it to understand the causes of variation and therefore optimize its performance. The organization will be able to show that continuous process improvement is being enabled by quantitative feedback from its embedded processes and from validating innovative ideas and technologies. The organization will be able to demonstrate a strong alignment of objectives with business plans, and this will be cascaded down through scoping, sponsorship, commitment, planning, resource allocation, risk management and benefits realization. Top management is proactively seeking out innovative ways to achieve goals. Using metrics, management can effectively control processes and identify ways to adjust and adapt them to particular initiatives without loss of quality.

(E) The organization is focused on the optimization of its quantitatively managed processes

to take into account changing business needs and external factors. It can

anticipate future capacity demands and capability requirements to meet delivery

challenges – e.g. through portfolio analysis. Top managers are seen as exemplars, reinforcing the need and potential for capability and performance improvement. The knowledge gained by the organization from its process and product metrics will enable it to understand the causes of variation and therefore optimize its performance. The organization will be able to show that continuous process improvement is being

enabled by quantitative feedback from its embedded processes and from validating

innovative ideas and technologies. The organization will be able to demonstrate

a strong alignment of organizational objectives with business plans, and this will

be cascaded down through the scoping, sponsorship, commitment, planning, resource

allocation, risk management, and benefits realization.

**Question 2**. Our management control is best described by:

(A) Project management terminology is used by some members of the organization but
not consistently and possibly not understood by all stakeholders. Projects are
conducted and managed according to individual preferences.

(B) The concepts of project management will have been grasped by the organization, and there may be local experts, such as experienced project managers, working on key
projects.

(C) There is a centrally defined and documented approach to a project management life

cycle and controls, and it is applied in all projects by capable staff who support

project teams.

(D) Project management is seen as a key tool for the delivery mechanism of change.

Within the project environment, the focus is on the improvement of delivery through

measurement and analysis of performance.

(E) Management controls can ensure that the project approach delivers the change objectives

of the organization. Acceptance of project management as the optimal approach to

change delivery is organization-wide. There is evidence of continual improvement.

**Question 3.** Our benefits management is best described by:

(A) There is some recognition that the concept of benefits can be differentiated from

project outputs.

(B) Benefits are recognized as an element within project business cases. There may be

some documentation regarding who is responsible for particular benefits and their realization, but this is unlikely to be followed through or consistent.

(C) There is a centrally managed and consistent framework for defining and tracking the

realization of benefits arising from project outputs.

(D) Benefits management is embedded within the project management approach and

there is a focus on the delivery of business performance from project outputs. Project

performance metrics are collected and analyzed.

(E) Benefits management is embedded within the organizational approach to change and

is assessed as part of the development of an organizational strategy. Business

performance metrics are linked to and underpin, the recognition of benefits

realization. There is evidence of continual improvement.

**Question 4,** Our financial management is best described by:

(A) There is little or no financial control at the project level. There is a lack of accountability

and monitoring of project expenditure.

(B) Project business cases are produced in various forms and the better and more formal

cases will present the rationale on which to obtain an organizational commitment to the

project. The overall cost of the project is not monitored or fully accounted for.

(C) There are centrally established standards for the preparation of business cases and

processes for their management throughout the project life cycle. Project managers

monitor costs and expenditures per organizational guidelines and

procedures, with defined interfaces with other financial functions within the

organization.

(D) The organization can prioritize investment opportunities effectively in relation

to the availability of funds and other resources. Project budgets are managed

effectively and project performance against cost is monitored and compared.

(E) Project financial controls are fully integrated with those of the organization. Cost

estimation techniques used at the project level are continually reviewed in terms of

actual versus estimate comparisons to improve estimation throughout the

organization. There is evidence of continual improvement.

**Question 5.** Our approach to stakeholder engagement is best described by:

(A) Stakeholder engagement and communication is rarely used by projects as an element

of the delivery toolkit.

(B) Some projects will be communicated to stakeholders, but this is linked more to the

a personal initiative of project managers than a structured approach being deployed

by the organization.

(C) There is a centrally managed and consistent approach to stakeholder engagement and

communications used by all projects.

(D) Sophisticated techniques are used to analyze and engage the project stakeholder

environment effectively, and quantitative information is used to underpin the

assessment of effectiveness.

(E) Communications are being optimized from extensive knowledge of the stakeholder

environment, to enable the projects to achieve their objectives. There is evidence of

continual improvement.

**Question 6.** Our risk management is best described by:

(A) There is minimal evidence of risk management being used to any beneficial effect on

projects. There may be evidence of risks being documented but little evidence of

active management.

(B) Risk management is recognized and used on projects, but there are inconsistent

approaches that result in different levels of commitment and effectiveness.

(C) Project risk management is based on a centrally defined process that is cognizant of

the organization’s policy for the management of risks is used consistently.

(D) Project risk management is working effectively, is embedded, and the value of risk

management can be demonstrated. There is evidence of opportunity management and

management of risk aggregation.

(E) Risk management is embedded in the organizational culture and underpins all

decision-making within projects. There is evidence of continual improvement.

**Question 7.** We deliver organizational governance via:

(A) Some informal governance of projects exists but has undefined links to broader

organizational controls. Roles are unlikely to be formally defined.

(B) Project management from an organizational perspective is beginning to take shape

but with ad hoc controls and no clear strategic control. Roles and responsibilities will

be inconsistent, as will reporting lines.

(C) Centrally defined organizational controls are applied consistently to all projects, with

decision-making structures in place and linked to organizational governance.

(D) There will be aligned project decision-making processes that adapt and

integrate with broader organizational governance and which are transparent to those

involved. Project management responsibilities are embedded within broader role

descriptions.

(E) The governance arrangements for projects are a core aspect of organizational control,

with demonstrable reporting lines to the Executive Board level and with clear ownership

and control responsibilities embedded within the organization. There is evidence of

continual improvement.

**Question 8.** Our resource management is best described by:

(A) There is some recognition within the organization of the need to manage resources

effectively to enable successful delivery of projects, but little evidence of resource

acquisition, planning, or management.

(B) Resources are being deployed across the organization and individual projects have an

approach to resource acquisition, planning, or management. However, there is little

evidence of the consistency of the approach.

(C) The organization has a centrally defined and adopted set of procedures and

management processes for acquiring, planning, and managing project resources.

(D) Resource management for projects is considered at a strategic level within the

organization. There is evidence of resource capacity management, through capacity

planning, to meet project delivery needs.

(E) Resources are deployed optimally. There is clear evidence of load balancing and the

effective use of both internal and external resources across all projects. There is

evidence of continual improvement.

**Question 9.** The organization:

(A) Recognizes projects and runs them differently from its ongoing business. (Projects may

be run informally with no standard process or tracking system).

(B) Ensures that each project is run with its own procedures to a minimum

specified standard. (There may be limited consistency or coordination between

projects).

(C) Has its own centrally controlled project processes and individual projects can flex

within these processes to suit the particular project.

(D) Obtain and retain specific measurements on its project management performance and

run a quality management organization to better predict future performance.

(E) Undertake continuous process improvement with proactive problem and technology

management for projects to improve its ability to depict performance over

time and optimize processes.

This completes Section 1. Project Management Maturity Model (P3M3®) self-assessment

**Supplementary Tables**

**Table 1**. Number of Organizations in Each Maturity Level Based on Self-Assessment


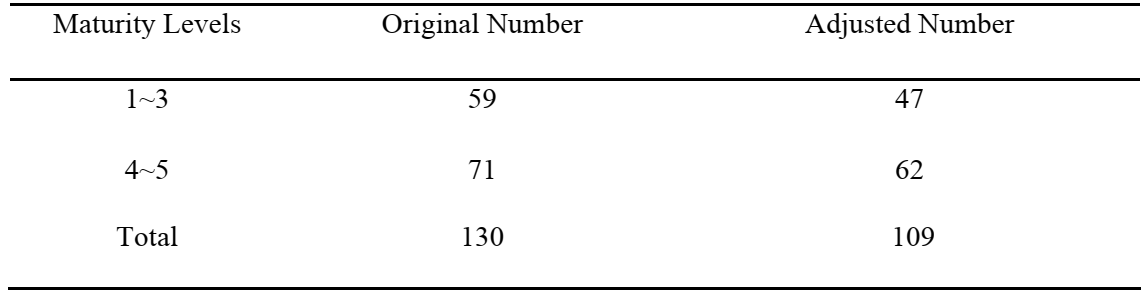
*Note*. Total number of responses =130. However, based on SPSS calculations, a further 21 data sets were excluded in the study based on incomplete responses. Therefore, n=109.

**Table 2.** Reliability Statistics

**
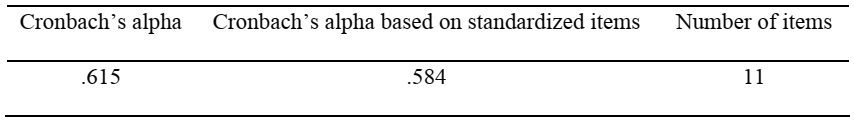
**

**Table 3.** Independent samples test *(p value)*

*
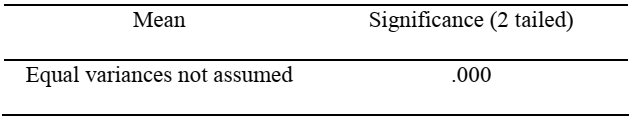
*


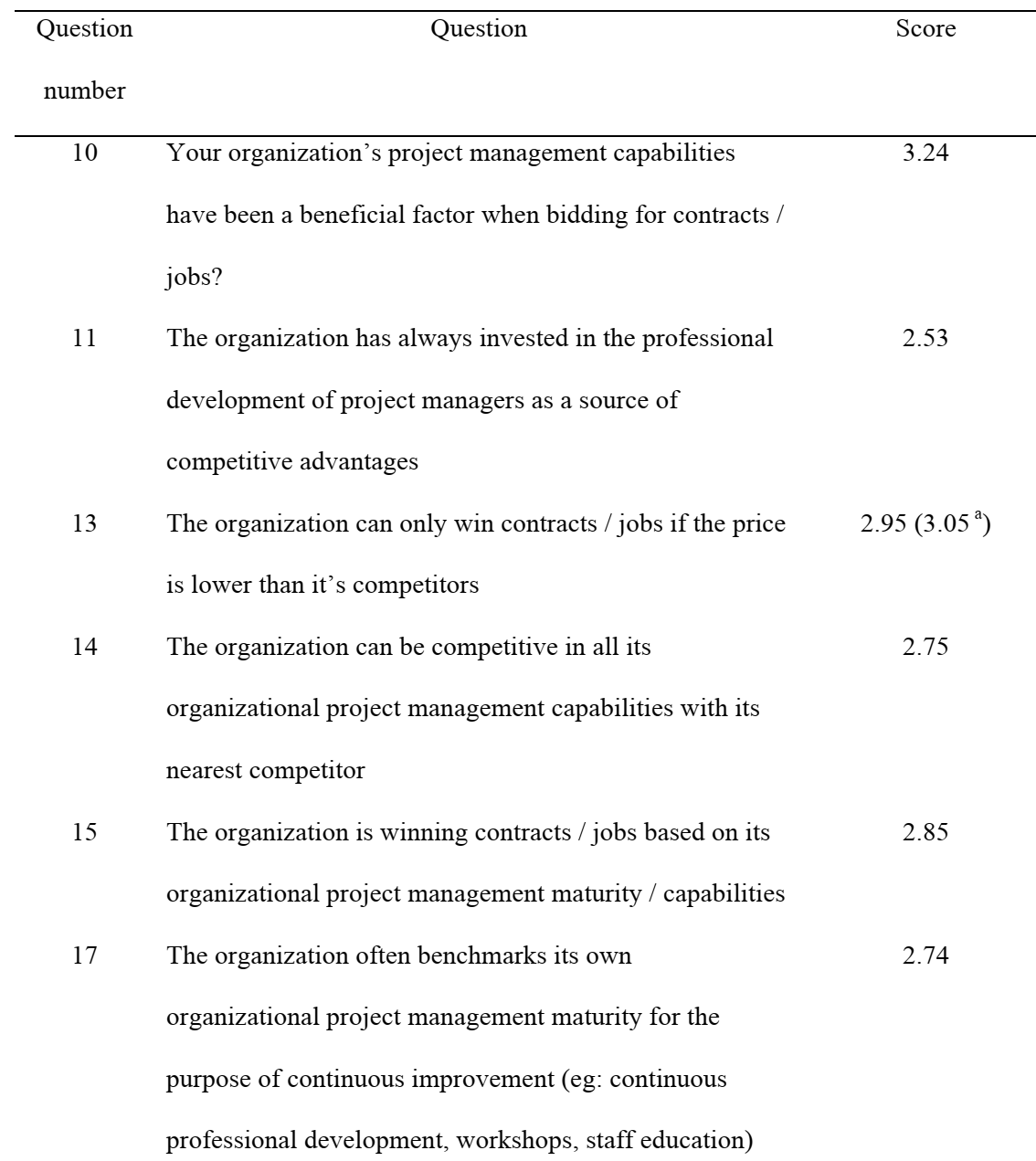

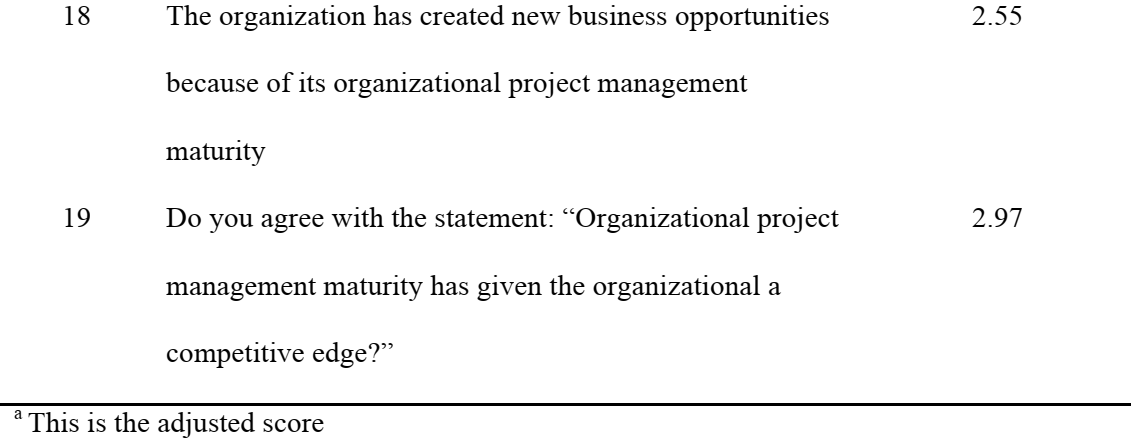
**Table 4.** Maturity Levels 1~3 Average Scores

**Table 5.** Maturity Levels 4~5 Average Scores


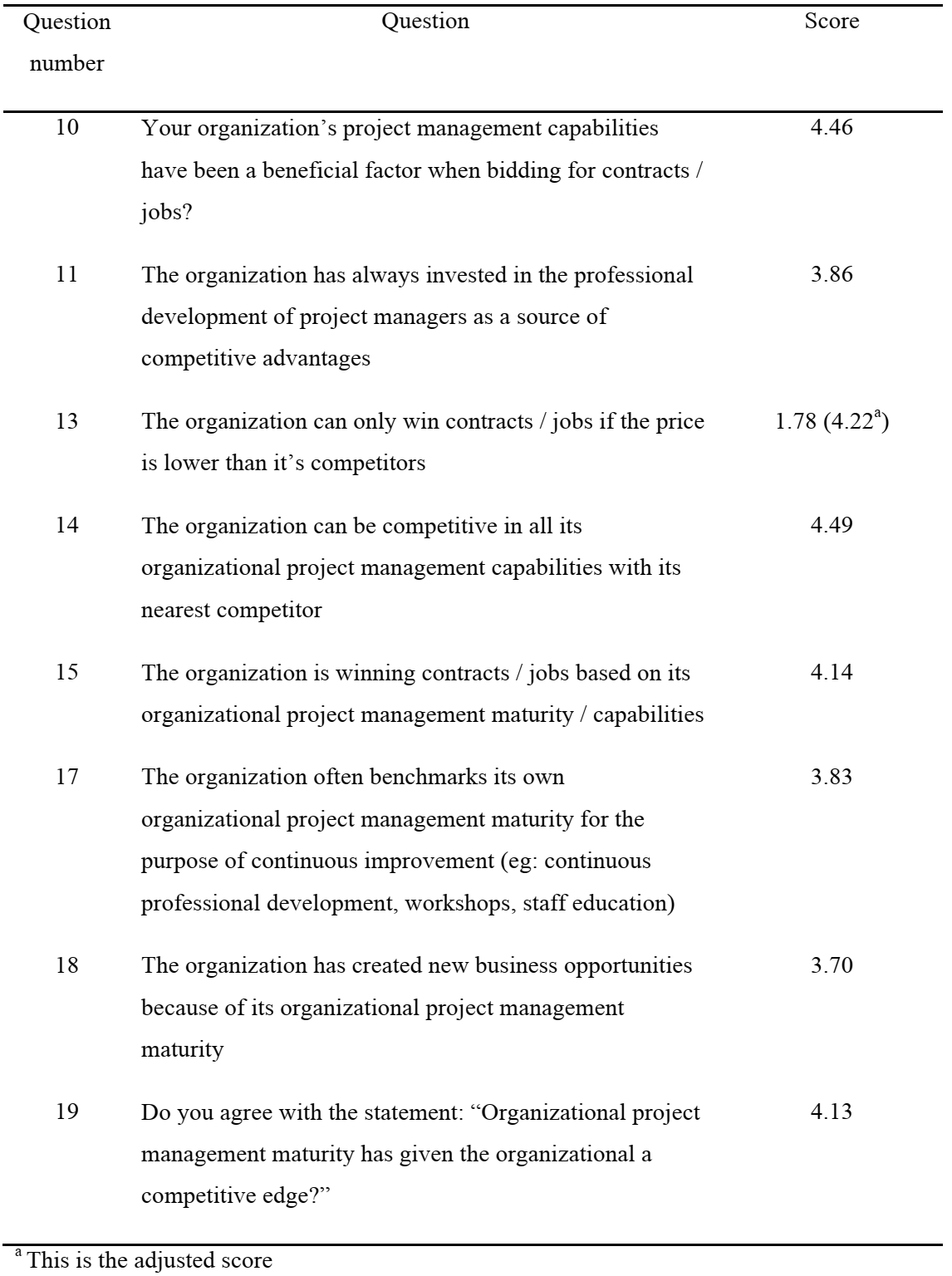


**Table 6.** Soft skills score


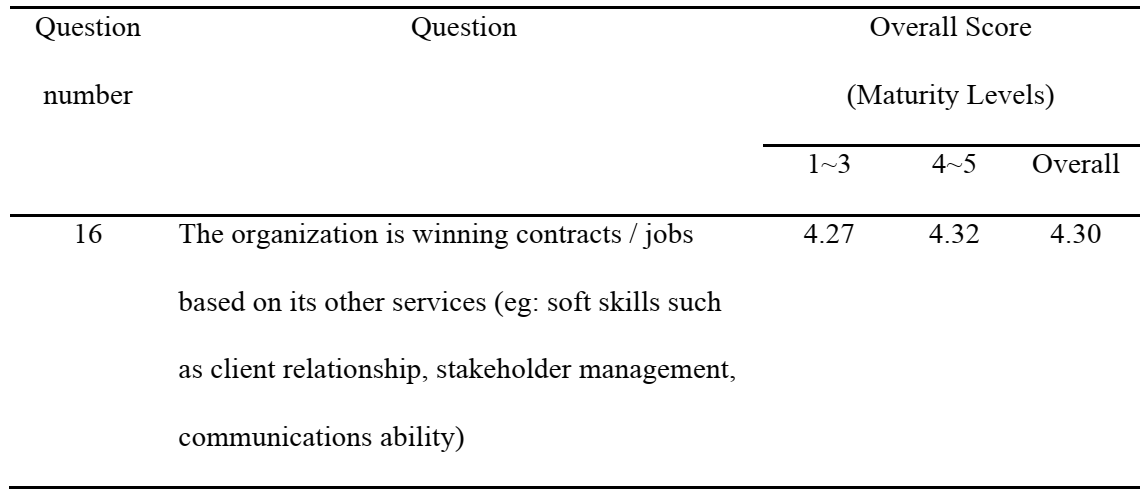

Supplement: S1 File — (DOCX) [file pone.0287225.s001.docx]
