# Supplementary material for: Competitive advantages of organizational project management maturity: A quantitative descriptive study in Australia
Source: PLoS One. 2023 Jun 27;18(6):e0287225. doi: 10.1371/journal.pone.0287225 (PMC10298749; doi:10.1371/journal.pone.0287225)
Supplement: Attachment — Submitted filename: Responses to Reviewers.docx [file pone.0287225.s002.docx]

Responses to Reviewers

We want to thank the reviewer for her time and valuable comments. Our editing and changes have been highlighted in red throughout the manuscript.

1. Introduction – 1st paragraph - project management is discipline used not only in “… information technology projects, building projects, new automotive products, airplanes, or weapons systems ...”. I recommend to modify it.

1. We have edited parts of the abstract that may have been confusing and corrected the misspelled word and also its reference format (added the year in the references section).

2. Introduction – 2nd paragraph – The skills of project managers could be certified. The mentioned certification from Australia is regional certification, I recommend to mention certifications used worldwide, not only Project Management Institute certification, but also PRINCE2 and International Project Management Association standards certifications.

2. We have added a short description of PRINCE2 in the introduction. We include in our study an Appendix (Appendix A) which lists over 20 different maturity models which suggest that project managers need to be proficient in their own competency in order to achieve maturity. We hope this can satisfy the reviewer’s concern.

3. Results – it will be useful to present in the article also description of answers of respondents. If they full filled self-assessment P3M3 questions it could be possible to present what is the level of project management maturity of respondent’s organizations. This information could give the overview about the maturity of project management. It could be important information, in case the maturity is mainly on low level (P3M3 has got 5 levels – from the lowest “awareness of process” to the highest “optimized process”) it could be the reason of the final statement: „organizational project management maturity has no effect on competitive advantages “. There would be very interesting the comparison of high-level project maturity organizations and low-level project maturity organizations – is there for both groups still the result same, or the level of project maturity could impact the relationship between organizational project management maturity and competitive advantages?

3. We added additional details of the responses’ results in the Results section to clarify our findings and to answer the reviewer’s points. We also added a new table (Table 2) and modified Table 3 by adding the results of maturity groups in it: 1) maturity levels 1 – 3 and 2) maturity levels 4 – 5 in order to clarify our findings and make it easier for the reader to understand them. Please see the red highlights in the results section.

4. References – there is possible to find many references but mainly to methodology of the research, to the main topic – project management maturity and competitive advantages – is not presented sufficient literature review.

4. We have added more details in the introduction, and also references to support the main topic. Please see the red highlights in the introduction (paragraphs 2 and 3, and references).

5. Supplementary material – there is presented the Project Management Maturity Model (P3M3®) self-assessment, but this project management maturity model is described in the literature, so it is sufficient just to offer to the readers the link to description of this model. The questions 10-20 are presented two times – in table and in supplementary material, it is redundant.

5. We have removed the redundant material/information in the supplementary material and added necessary links, appendixes, and tables. Please see the new revised the tables in the manuscript.
