# Supplementary material for: Competitive advantages of organizational project management maturity: A quantitative descriptive study in Australia
Source: PLoS One. 2023 Jun 27;18(6):e0287225. doi: 10.1371/journal.pone.0287225 (PMC10298749; doi:10.1371/journal.pone.0287225)
Supplement: Attachment — Submitted filename: Rebuttal Letter.docx [file pone.0287225.s003.docx]

Rebuttal Letter

We have addressed all of the concerns of the reviewer

Format comments

1. We have provided a title for Table 1. Please see the title in red highlights (revised manuscript)

Content comments

1. We have modified the Introduction paragraph as per reviewer’s suggestion. Please see the red highlights in paragraph 1.
2. We have added more content as per reviewer’s suggestion. Thank you for the great example and please see red highlights
3. We have separated the appendix into two parts as suggested by the reviewer. We now have appendixes A (project management maturity models) and B (General Maturity Models and Methods).
