# Supplementary material for: Competitive advantages of organizational project management maturity: A quantitative descriptive study in Australia
Source: PLoS One. 2023 Jun 27;18(6):e0287225. doi: 10.1371/journal.pone.0287225 (PMC10298749; doi:10.1371/journal.pone.0287225)
Supplement: Attachment — Submitted filename: Rebuttal Letter.docx [file pone.0287225.s004.docx]

Rebuttal Letter

We have addressed all of the concerns of the editorial team and reviewer

Editorial Team

1. We have moved the ethics statement into the beginning of the Methods section
2. We confirm that the manuscript and the attached Supporting Information file contains the minimal data set.

Reviewer

1. We have removed the cross reference #4 in the title page.
2. We have corrected the spelling error of Drouin (highlighted in red)
3. We have added a section in the Introduction titled Theoretical Background which includes references and/or literary sources within the last 5 years as per reviewer’s suggestion. Thank you for your details (please see the red highlights).
4. We have provided more details of the methodology as per reviewer’s suggestion (please see red highlights in the Methods section).
